# Indispensable Roles of Plastids in *Arabidopsis thaliana* Embryogenesis

**DOI:** 10.2174/138920210791616716

**Published:** 2010-08

**Authors:** Shih-Chi Hsu, Mark F Belmonte, John J Harada, Kentaro Inoue

**Affiliations:** 1 Department of Plant Sciences, University of California, Davis, CA, USA; 2 Department of Plant Biology, University of California, Davis, CA, USA; 3 Department of Biological Sciences, Faculty of Science, University of Manitoba, Winnipeg, Canada

**Keywords:** Arabidopsis thaliana, embryogenesis, globular embryo, microarray, plastid, preglobular embryo, SeedGenes.

## Abstract

The plastid is an organelle vital to all photosynthetic and some non-photosynthetic eukaryotes. In the model plant *Arabidopsis thaliana*, a number of nuclear genes encoding plastid proteins have been found to be necessary for embryo development. However, the exact roles of plastids in this process remain largely unknown. Here we use publicly available datasets to obtain insights into the relevance of plastid activities to *A. thaliana *embryogenesis. By searching the SeedGenes database (http://www.seedgenes.org) and recent literature, we found that, of the 339 non-redundant genes required for proper embryo formation, 108 genes likely encode plastid-targeted proteins. Nineteen of these genes are necessary for development of preglobular embryos and/or their conversion to globular embryos, of which 13 genes encode proteins involved in non-photosynthetic metabolism. By contrast, among 38 genes which are dispensable for globular embryo formation but necessary for further development, only one codes for a protein involved in metabolism. Products of 21 of the 38 genes play roles in plastid gene expression and maintenance. Examination of RNA profiles of embryos at distinct growth stages obtained in laser-capture microdissection coupled with DNA microarray experiments revealed that most of the identified genes are expressed throughout embryo morphogenesis and maturation. These findings suggest that metabolic activities are required at preglobular and throughout all stages of embryo development, whereas plastid gene expression becomes necessary during and/or after the globular stage to sustain various activities of the organelle including photosynthetic electron transport.

## INTRODUCTION

Plastids are organelles derived from an ancient form of cyanobacteria by endosymbiosis [1] and are vital for all photosynthetic and some nonphotosynthetic eukaryotes. In higher plants, plastids are present in all cell types except male gametophytes of certain species [2, 3]. Plastids exist in several distinct forms, such as chloroplasts in photosynthetic tissues, chromoplasts in yellow, orange, and some red fruits and flower petals, amyloplasts in non-colored storage tissues, and undifferentiated proplastids in meristematic cells. Most of these plastids are inter-convertible, and their development is closely associated with plant growth and development [4]. In addition to the oxygenic photosynthetic activity of chloroplasts, numerous metabolic processes such as the biosynthesis and accumulation of starch, lipids, amino acids, and various isoprenoids, including carotenoids and precursors to gibberellins, take place in plastids [5-8]. Hence, properly-functioning plastids are essential for the viability of plants, although this idea has not been systematically addressed. During the evolution of plastids, most of the genes in the cyanobacterial endosymbiont have been transferred to the host nuclear genome [1]. The resultant plastid still contains its own genome, which encodes about 100 proteins including major components of the photosynthetic electron transport machineries and the large subunit of ribulose 1,5-bisphosphate carboxylase/oxygenase [9]. However, most plastid proteins are encoded in the nuclear genome, and the majority of these proteins are synthesized on cytoplasmic ribosomes as a precursor with an N-terminal extension called the transit peptide. Transit peptide-dependent protein import across the double-membrane envelope of plastids is catalyzed by two distinct protein complexes in the outer and inner membranes called TOC and TIC (Translocon at the Outer and Inner-envelope membrane of Chloroplasts), respectively [10]. Based on extensive evaluation of several prediction programs that identify proteins with a transit peptide, a total of 2,100 nuclear genes were predicted to encode plastid proteins in the model plant *Arabidopsis thaliana *[11]. Furthermore, no more than 100 plastid proteins encoded by nuclear genes are synthesized without a transit peptide; they include most outer envelope proteins [12], a few inner envelope proteins [13, 14] and α-carbonic anhydrase that is sorted through a secretory pathway [15]. 

**Fig. (1). Overview of terminal phenotype classification of SeedGenes and microarray analyses on embryo development.**
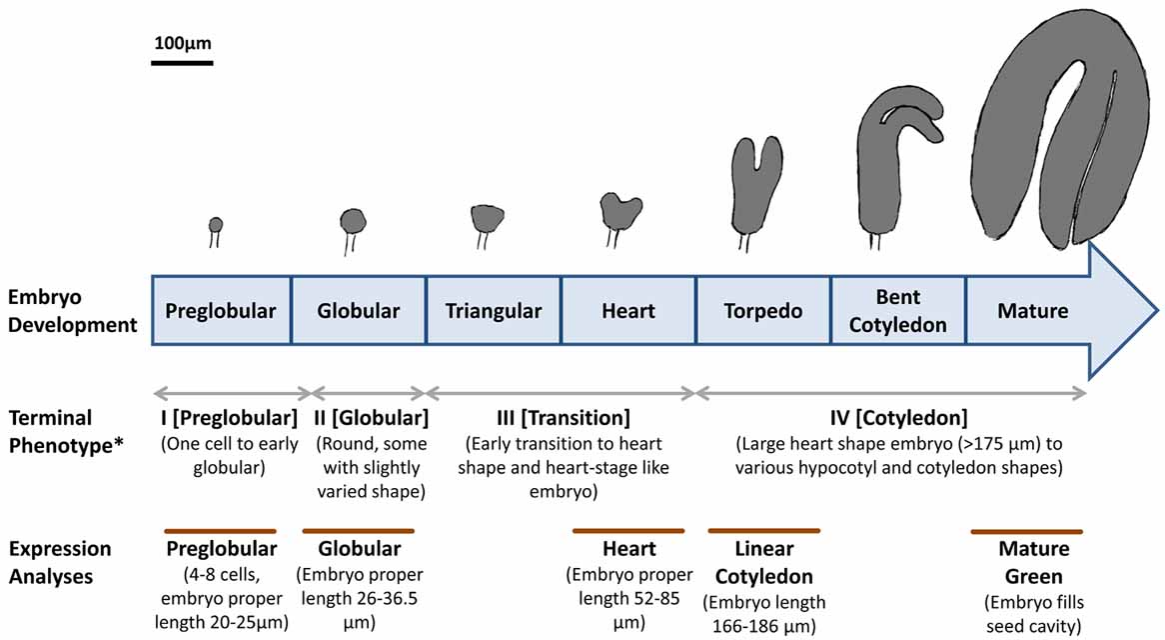
A series of embryo development stages are listed in different boxes in the arrow (from left to right: early to late stages) and corresponding embryos (approximately to scale) are shown above the arrow. The stages at which embyos were taken for laser capture microdissection and microarray analyses (http://seedgenenetwork.net) are listed below the arrow and indicated by brown lines. Gene Expression Omnibus Accession numbers of the data are: GSE11262, 12403, 12404, 15160, and 15165. The terminal phenotypes of embryo-defective mutants were defined by SeedGenes (http://www.seedgenes.org). ^*^According to SeedGenes database, mutant embryos were removed from seeds prior to desiccation and examined under a dissecting microscope. Seeds classified as I [preglobular] often contain an early globular embryo too small to be seen upon seed dissection. These early globular embryos can be seen using Nomarski optics. (For interpretation of the references to color in this figure legend, the reader is referred to the web version of this paper).

In the life cycle of flowering plants, embryogenesis is a crucial developmental period, which can be divided into two distinct phases [16]. The first phase is morphogenesis during which the basic body plan of the plant is established. The second is the maturation phase that involves cell growth and expansion, and accumulation of macromolecules that promote tolerance to the desiccation period and seedling growth. Embryo morphogenesis begins with the single-celled zygote which, in *A. thaliana*, undergoes a stereotypical cell division pattern giving rise to preglobular, globular, heart, torpedo, linear cotyledon, bent-cotyledon, and mature green stage embryos. Undifferentiated plastids begin to develop into chloroplasts and increase their numbers at the torpedo stage before embryos enter into the maturation phase (Fig. **1**) [17]. At the maturation phase, storage products such as starch, lipid and proteins accumulate in the embryo in preparation for a period of metabolic quiescence and developmental arrest. Embryos resume development as seedlings when the appropriate environmental conditions are met, and seeds germinate. Molecular genetic studies have identified genes encoding proteins involved in controlling nuclear gene expression and auxin transport as key embryonic regulators in *A. thaliana *[18]. However, our understanding of the molecular mechanisms underlying seed development of this model plant is not complete. Functional genomics provides information that can be used to better understand the molecular basis for embryo development. Several projects with data publicly available are underway, such as the “Gene Networks in Seed Development project” (http://seedgene-network.net), which utilizes laser capture microdissection, microarray and high-throughput sequencing technologies to profile the mRNA sets present in different seed regions and compartments throughout development (John J. Harada, unpublished). Another example is the “SeedGenes project” (http://www.seedgenes.org), which presents comprehensive information about *A. thaliana *genes that are essential for seed development [19, 20].

A cytological study showed that plastids in *A. thaliana* embryonic cells remain as undifferentiated non-photosynthetic forms without detectable starch accumulation until the late globular stage when grana become visible [17]. Although the exact roles of these plastids remain unclear, a number of nuclear genes encoding plastid proteins have been found to be required for embryogenesis (see below). We are interested in elucidating roles of plastids vital for various stages of plant development. In this article, we make use of publicly available datasets to shed light on the relevance of plastid activity to plant embryogenesis.

## IDENTIFICATION OF NUCLEAR GENES ENCODING PLASTID PROTEINS NECESSARY FOR EMBRYOGENESIS IN *ARABIDOPSIS THALIANA*

The SeedGenes database (Release 7, December, 2007) [20] lists 358 genes that give a mutant seed phenotype when disrupted by mutation. Knockout mutations of 323 genes cause arrests at various stages of embryo development. Seeds of some mutants showing an arrest phenotype at the late stage of embryo morphogenesis (cotyledon stage) can germinate and sometimes develop into mature plants (e.g., [21]). The SeedGenes database includes corresponding genes because they are needed for normal growth and development of seeds [22]. Since the latest release of SeedGenes, an additional 16 genes have been reported to be necessary for embryo development in *A. thaliana* [23-36], making the total number of genes known to be required for embryogenesis 339. This number corresponds to about 30-60% of all the genes necessary for proper embryo development in this model species based on previous estimates [22, 37]. 

Null-mutants of most of these genes are arrested at a single stage. However, in some cases, a single null mutation causes embryos to arrest at a wide range of developmental stages (e.g., [38]). It has also been shown that different null mutant alleles of a single gene can result in different terminal phenotypes (e.g. [39]; SeedGenes Database). These findings may indicate that a gene is required at the beginning of a certain embryonic stage but the mutation does not immediately cause an arrest of development. Alternatively, the mutation may only indirectly affect embryogenesis, having a primarily effect in a seed compartments other than embryos [38]. 

**Fig. (2). Flow chart indicating the identification of Arabidopsis thaliana genes encode plastid proteins indispensable for embryogenesis.**
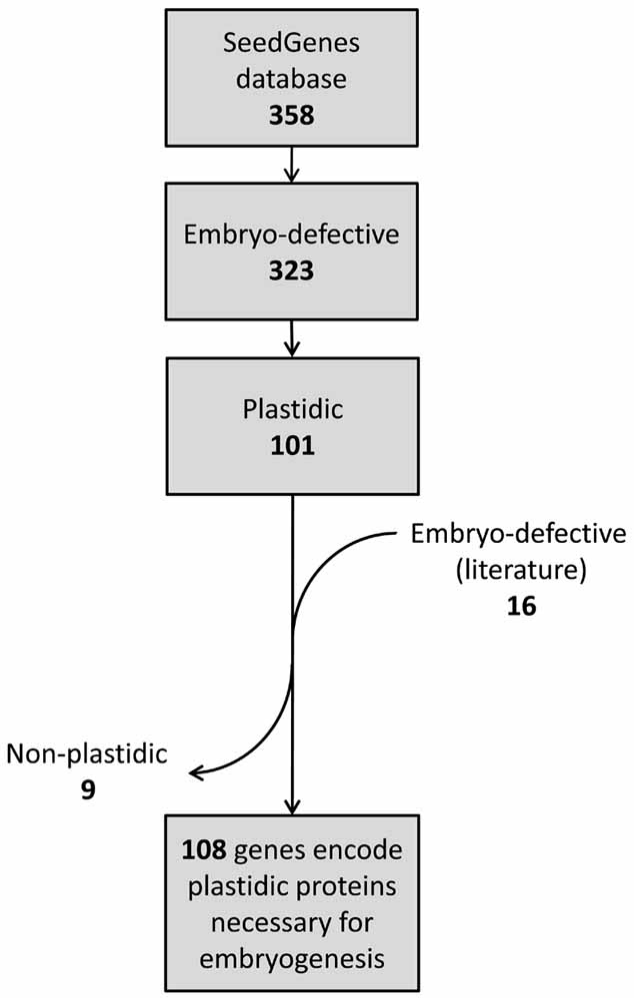
The SeedGenesdatabase (http://www.seedgenes.org; last updated December, 2007) contains 358 *A. thaliana* genes that give a seed phenotype when disrupted by mutation. Among these genes, 323 of them are necessary for embryogenesis and their disruption results in arrests in development. To determine the localization of encoded proteins, three approaches were used: literature search, Plant Proteome Database (PPDB) search, and compurter algorithm prediction (TargetP). Literature search also revealed that 16 additional genes are necessary for embryogenesis and 7 of them encode plastid proteins, resulting in a total of 108 non-redudant genes necessary for embryogenesis.

By a thorough search of the available literatures and the Plant Proteome DataBase [40], and also by using a computer prediction program to detect transit peptides [41], we estimated that 101 out of 323 genes in the SeedGenes database and seven of the 16 recently reported genes most likely encode proteins targeted to plastids (Fig. **2**; Table **1**). Hence, 108 out of 339, or about one third of non-redundant genes necessary for *A. thaliana *embryogenesis encode plastid proteins. This fraction is about three times larger than the proportion in *A. thaliana *nuclear genes encoding plastid-targeted proteins, which include proteins with a transit peptide (8%; [11]) and those without (less than 1%: including most outer envelope proteins [12], two inner envelope proteins [13, 14] and α-carbonic anhydrase [15]). This apparent overrepresentation of genes encoding plastid proteins may suggest that functional plastids are required for normal embryo development [20]. However, we cannot completely exclude a possibility that availability of embryo-defective mutants may be skewed toward genes encoding plastid proteins for some unknown reasons. Genome-wide bioinformatics analyses are necessary to address these possibilities. Recently, 122 independent lines with mutations in nuclear genes encoding plastid proteins were reported from *A. thaliana* as potential embryo-lethal mutants based on the lack of viable homozygous mutants [42]. Interestingly, among the 91 genes represented by these lines, only 16 genes are found in our list (Table **1**). It remains to be determined whether the inability to obtain viable homozygous mutants corresponding to the other 76 genes is due to embryo-lethality. 

## FUNCTIONAL DISTRIBUTION OF PLASTID PROTEINS ENCODED BY GENES REQUIRED FOR VARIOUS STAGES OF EMBRYO DEVELOPMENT

We next put each of the identified genes into one of the four groups based on the reported terminal phenotype of the null mutants (arrested at preglobular (I), globular (II), transition of globular to heart (III), and cotyledon stages (IV), respectively; Fig. **1**) and also into one of six categories (metabolism, gene maintenance and expression, protein trafficking, protein homeostasis, membrane transport, and unknown) based on functions of their products as demonstrated by published studies and/or annotated in the publicly available databases (Table **1**). For a gene with a single mutant allele showing heterogeneous seed phenotypes, or the one with multiple alleles showing different phenotypes, the earliest stage was used for grouping because we consider that it should be the stage when the gene is first required. 

**Fig. (3). Functional grouping of genes encoding plastid proteins essential for A. thaliana embryogenesis.**
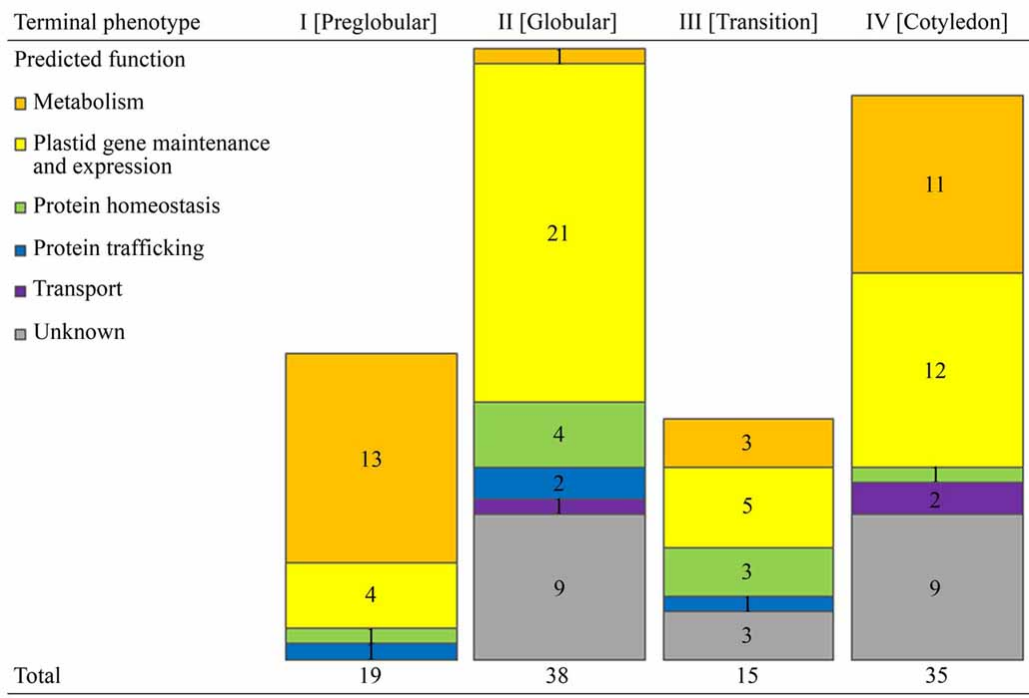
TGenes essential for *A. thaliana* embryogenesis and encoding plastidic proteins are grouped by their mutant phenotypes and predicted functions. Predicted functions are based on sequence comparison and/or experimental data, and divided into six categories: metabolism (orange blocks), plastid gene maintence and expession (yellow blocks), protein homeostasis (green blocks), protein trafficking (blue blocks), transport (purple blocks), and unknown (gray blocks). (For interpretation of the references to color in this figure legend, the reader is referred to the web version of this paper).

As shown in Fig. (**3**), our analysis revealed a clear shift in functionalities necessary at two early stages of embryo development. Group I consists of 19 genes, which are required for proper development of preglobular embryos and/or their conversion to globular embryos. Among them, 13 genes encode enzymes, including those responsible for the biosynthesis of acetyl-CoA, histidine, nicotinamide adenine dinucleotide, and folate, four code for proteins related to plastid DNA replication, transcription, and translation, and two others encode a precursor protein import channel (Toc75) and a molecular chaperone (GrpE) (Table **1**). Group II consists of 38 genes which are dispensable for globular embryo formation but become necessary for further embryo morphogenesis and maturation. By contrast to Group I, Group II is enriched with genes encoding proteins involved in organellar gene expression and maintenance, such as pentatricopeptide repeat-containing proteins, tRNA synthetases, and ribosomal proteins (Table **1**). Only one gene in Group II encodes a protein in the metabolism category, and its product is responsible for a later step of histidine biosynthesis [36]. Groups III and IV, which includes a total of 15 and 35 genes, respectively, are more diverse than the former two in functional categories (Fig. **3**; Table **1**). The clear functional differences between the first two groups of genes may be due to the necessities of operating basic metabolic pathways from a very early stage of embryogenesis, and/or producing a massive amount of proteins encoded by the plastid genome at the globular stage.

## EXPRESSION PATTERN OF GENES ENCODING PLASTID PROTEINS REQUIRED FOR EMBRYOGENESIS AT DIFFERENT STAGES OF SEEDS

To examine whether the plastid-protein-encoding genes we identified are expressed in embryos, we analyzed seed RNA profiles from DNA microarray experiments. The samples used for RNA extraction were captured from seven distinct seed compartments at five developmental stages obtained by laser capture microdissection, assuring precise sampling without contamination from adjacent compartments (Fig. **1**). Among the 108 genes corresponding to embryo lethal mutations that encode plastid proteins (Table **1**), unambiguous data for 95 genes were available (Table **2**). Data for the rest of 13 genes were unavailable or ambiguous although one gene in this group was reported to be expressed in embryo (Table **3**). Of the group of 95 genes, expression of eighty-one genes was confirmed, whereas expression of eight genes was under the detection limit of microarrays in any of the seed compartments. Interestingly, six other genes were not detectably expressed in embryos, but they were expressed in at least one of the other seed compartments (Table **4**). It is possible that their functions in compartments other than embryos are required for proper embryo development, similar to previously reported cases [43, 44]. 

**Fig. (4). Expression pattern of gene encoding plastid proteins necessary for A. thaliana embryogenesis.**
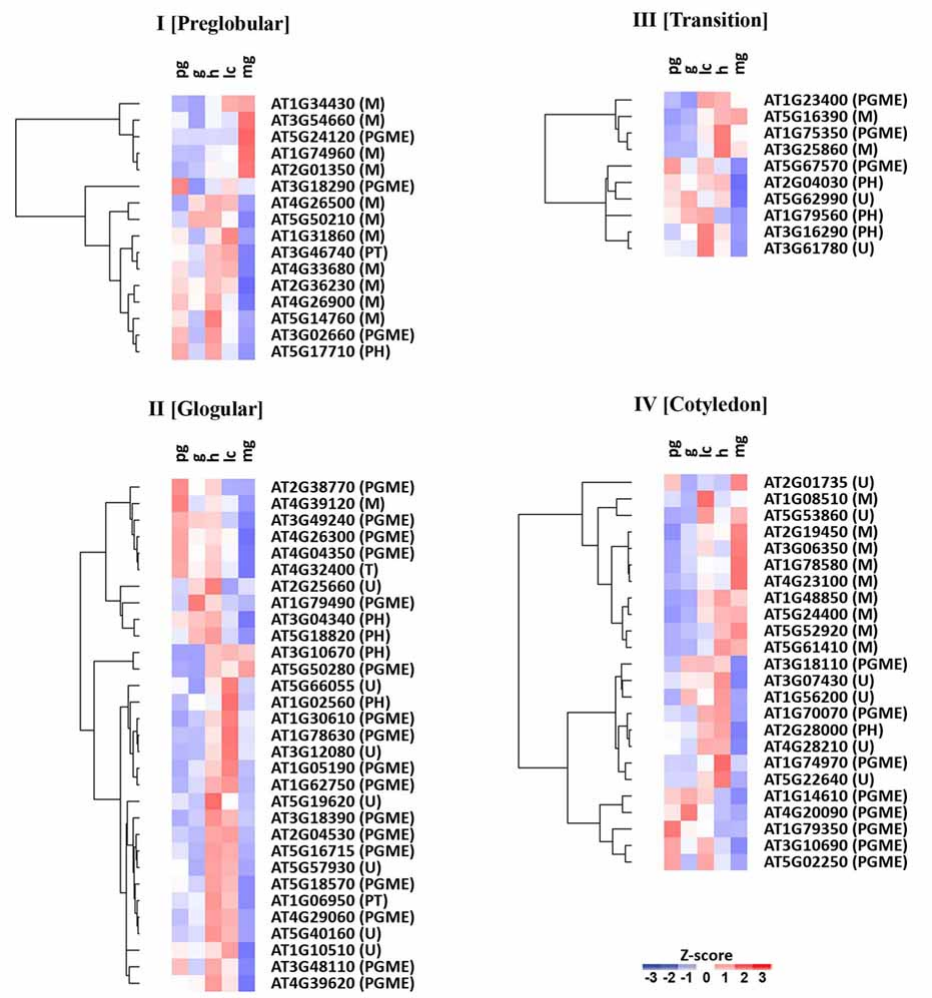
Heat map showing the variation in levels (Z-score) of the indicated mRNAs encoding plastid proteins in embryos at different stages of development (columns: pg, preglobular; g, globular; h, heart; lc, linear cotyledon; mg, matrue green). Predicted functions of gene products are indicated in parentheses (M, metabolism; PGME, plastid gene maintenance and expression; PH, protein homeostasis; PT, protein trafficking; T, transport; U, unknown). Genes with an expression under the detection limit at all five stages were not included in the analysis.

Within the group of 81 genes expressed in embryos, 56 genes are expressed at five distinct embryonic stages. We wondered if a gene is most highly expressed at the stage at which the corresponding mutant is arrested. However, no obvious correlation between expression level and terminal phenotype was observed with the possible exception of genes in Group IV. Approximately one-half of the genes that are necessary at the cotyledon stage are highly expressed in linear cotyledon-stage embryos (Fig. **4**).

## CONCLUSIONS

As an endosymbiotic organelle, the plastid shares various properties with its prokaryotic relatives, the cyanobacteria. The plastids of higher plants have also gained the ability to develop into a variety of non-photosynthetic types and play vital roles in the growth and development of the organisms. However, plastid functions that are essential at each developmental stage are not known, except for chloroplasts in photosynthetic tissues. The current work takes an *in silico* approach to shed light on the functions of plastids during embryogenesis, the earliest stage of plant development following zygote formation. Although the analysis was limited to a set of non-redundant genes, our findings suggest that the non-photosynthetic metabolic activities of plastids is a prerequisite for the transition of preglobular to globular embryos and that the requirement for proteins involved in plastid gene expression becomes significant at or after the globular stage. Furthermore, analysis of the microarray data confirmed expression of most of these genes in the embryos. Based on these results, we hypothesize that i) the early stage of embryogenesis (from preglobular to globular) requires metabolic activities of plastids which are critical for various cellular processes possibly including those known to be essential for embryo development, i.e., cell division, nuclear gene expression, and auxin transport, and ii) activation of plastid gene expression that establishes various organelle activities including photosynthetic electron transport becomes necessary for the later stage of morphogenesis (from globular to heart), prior to when embryos start preparing for maturation. Furthermore, the current work poses several interesting questions. What are the effects of embryo-lethal mutations on the morphology of plastids? Apparently, some components of plastid gene expression and maintenance are not required for the formation of globular embryos. Does this mean that the organelle gene expression is not necessary at all until this stage of embryo morphogenesis? Development of strategies that allow visualization and morphological examination of aborted seed plastids, and also additional genetic and biochemical studies are needed to test these hypotheses and questions.

## Figures and Tables

**Table 1.** Nuclear Genes Encoding Plastid Proteins Required for *Arabidopsis thaliana* Embryogenesis

Gene^a^	ID^b^	Function (Cat)^c^		References^d^		
				EMB	F	L
**I. Genes whose disruption causes embryo abortion at preglobular stage (19)**						
At1g34430	NC	dihydrolipoamide S-acetyltransferase^#^	(M)	S	[45]	P
At4g33680	C	aminotransferase class I and II family protein^#^	(M)	[46]	[46]	[46]
At1g74960	C	ketoacyl-acyl carrier protein synthase^#^	(M)	[47]	–	P
At4g26900	C	imidazole glycerol phosphate synthase^#^ (His biosynthesis)	(M)	[48]	–	P
At2g36230	C	N'-5'-phosphoribosyl-formimino-5-aminoimidazole-4-carboxamide ribonucleotide isomerase^#^ (His biosynthesis)	(M)	[48]	[49]	P
At1g31860	C2’	phosphoribosyl-ATP pyrophosphohydrolase/phosphoribosyl-AMP cyclohydrolase (His biosynthesis)	(M)	[48]	–	P
At5g10330^+^	C	histidinol phosphate aminotransferase^#^ (His biosynthesis)	(M)	[48]	–	P
At5g14760	C	L-asp oxidase (NAD biosynthesis)	(M)	[50]	[50]	[50]
At5g50210^+^	C	ouinolinate synthase (NAD biosynthesis)	(M)	[50]	[50]	[50]
At2g01350	C	ouinolinic acid phosphoribosyl transferase (NAD biosynthesis)	(M)	[50]	–	[50]
At2g28880	NC	4-amino-4-deoxychorismate synthase (folate biosynthesis)	(M)	S	[51]	[51]
At3g54660	C2	glutathione reductase^#^	(M)	S	[52]	[52]
At4g26500	C	activator of plastidic and mitochondrial desulfurases (AtSufE)	(M)	[53]	[53]	[53]
At1g08840	C2	DNA replication helicase^#^	(PGME)	S	–	T
At5g24120	C	RNA polymerase sigma subunit SigE^#^	(PGME)	[54]	–	[55]
At3g02660	C	tyrosyl-tRNA synthetase^#^	(PGME)	[56]	–	[57]
At3g18290	C2’	zinc finger protein-related^#^	(PGME)	S	–	T
At5g17710	C	co-chaperone GrpE family protein^#^	(PH)	S	–	P
At3g46740^+^	C	precursor protein import channel (Toc75)	(PT)	[58, 59]	[58, 59]	[58]
**II. Genes whose disruption causes embryo abortion at globular stage (38)**						
At4g39120^*^	C	histidinol-phosphate phosphatase (His biosynthesis)	(M)	[36]	[36, 60]	[36]
At2g01860	C	pentatricopeptide (PPR) repeat-containing protein^#^	(PGME)	S	–	T
At5g03800	C	similar to pentatricopeptide (PPR) repeat-containing protein^#^	(PGME)	[61]	–	T
At1g30610^+^	C	pentatricopeptide (PPR) repeat-containing protein^#^	(PGME)	[61]	–	T
At1g79490	NC	pentatricopeptide (PPR) repeat-containing protein^#^	(PGME)	S	–	T
At4g39620	C	pentatricopeptide (PPR) repeat-containing protein^#^	(PGME)	[61]	–	T
At5g50280	NC	pentatricopeptide (PPR) repeat-containing protein^#^	(PGME)	S	–	T
At3g49240	C	pentatricopeptide (PPR) repeat-containing protein^#^	(PGME)	[61]	–	P
At2g38770	C	U5 associated protein	(PGME)	S	–	P
At5g26742	NC	DEAD/DEAH box RNA helicase^#^	(PGME)	S	–	P
At3g18390^+^	NC	chloroplast splicing factor (CRS1)	(PGME)	S	–	P
At4g26300	NC	arginyl-tRNA synthetase^#^	(PGME)	[56]	–	[57]
At1g05190^+^	NC	ribosomal protein L6 family protein^#^	(PGME)	S	–	P
At1g78630	NC	ribosomal protein L13 family protein^#^	(PGME)	S	–	P
At4g04350	C	leucyl-tRNA synthetase^#^	(PGME)	[56]	–	[57]
At1g62750^+^	C	elongation factor Tu family protein^#^	(PGME)	[62]	–	[63]
At2g04842	NC	threoninyl-tRNA synthetase^#^	(PGME)	[56]	–	[57]
At5g16715	C	valyl-tRNA synthetase^#^	(PGME)	[56]	–	[57]
At4g29060	C	elongation factor Ts family protein^#^	(PGME)	S	–	P
At3g48110^+^	C2	glycyl-tRNA synthetase^#^	(PGME)	[56]	–	[57]
At2g04530^*^	NC	RNase Z^#^	(PGME)	[27]	–	[27]
At5g18570^*^+	C	GTP1/OBG family protein^#^	(PGME)	[32]	–	[32]
At3g10670	C	plastidic SufC-like protein (Fe-S cluster biogenesis)	(PH)	[64]	[64]	[64]
At3g04340^+^	NC	FtsH protease family protein^#^	(PH)	S	–	P
At5g18820	NC	RuBisCO subunit binding-protein alpha subunit (Cpn60a)^#^	(PH)	S	–	T
At1g02560^*^	C	ATP-dependent Clp protease proteolytic subunit (ClpP5)^#^	(PH)	[28]	[28]	[65]
At1g06950^+^	C	chloroplast protein import (Tic110)	(PT)	[66]	[66]	[67]
At2g31530	C	secY family protein^#^	(PT)	S	–	T
At4g32400^*^	C	nucleotide export	(T)	[33]	[68]	[68]
At5g19620^*^	C	OEP80	(U)	[35]	–	[69]
At5g66055^+^	C	ankyrin repeat protein (AKRP)^ #^	(U)	[70]	–	[70]
At1g10510	C	leucine-rich repeat family protein, similar to ribonuclease inhibitor^#^	(U)	S	–	P
At3g12080	C	GTP-binding family protein^#^	(U)	S	–	P
At5g63420	C	metallo-beta-lactamase family protein^#^	(U)	S	–	P
At3g24560	C	ATP binding	(U)	[71]	–	T
At5g40160	C	ankyrin repeat protein^#^	(U)	[72]	[73]	[70]
At2g25660	C	unknown	(U)	S	–	T
At5g57930	C	Fe-S cluster related	(U)	S	–	T
**III. Genes whose disruption causes embryo abortion at the transition of globular to cotyledon stage (15)**						
At3g25860	C	dihydrolipoamide S-acetyltransferase^#^	(M)	S	[74]	[74]
At5g16390	C	biotin carboxyl carrier protein of acetyl-CoA carboxylase^#^	(M)	S	[75, 76]	[76]
At3g20440	C2	1,4-alpha-glucan branching enzyme^#^ (starch biosynthesis)	(M)	S	–	P
At5g67570	C4	pentatricopeptide (PPR) repeat-containing protein^#^	(PGME)	S	–	[77]
At3g29290	NC	pentatricopeptide (PPR) repeat-containing protein^#^	(PGME)	S	–	T
At1g75350	NC	ribosomal protein L31 family protein^#^	(PGME)	S	–	P
At5g22800	C2	aminoacyl-tRNA synthetase^#^	(PGME)	[56]	–	[57]
At1g23400	C2	chloroplast intron splicing factor	(PGME)	[78]	[78]	P
At2g04030^+^	C	heat shock protein (Hsp90)	(PH)	S	[79, 80]	[79]
At1g79560	C2’	AAA and metalloprotease (FtsH12)	(PH)	S	–	[81]
At3g16290	C2	FtsH protease (AAA ATPase)	(PH)	S	–	P
At4g23430	C	subunit of Tic complex (Tic32), short chain dehydrogenase	(PT)	[82]	–	[82]
At5g62990	C	unknown	(U)	S	–	T
At3g61780	C2	unknown	(U)	S	–	T
At2g37920	NC	copper transporter related	(U)	S	[83]	T
**IV. Genes whose disruption causes embryo abortion at cotyledon stage (35)**						
At4g30580	C	2-acylglycerophosphoethanolamine acyltransferase	(M)	[84]	[85]	[84]
At1g48850	NC	chorismate synthase/5-enolpyruvylshikimate-3-phosphate phospholyase^#^	(M)	S	–	P
At3g55610	C	delta 1-pyrroline-5-carboxylate synthetase B^#^ (Pro synthesis)	(M)	[86]	–	[86]
At5g61410	C	ribulose-5-phosphate-3-epimerase^#^	(M)	S	–	P
At3g06350	C	dehydroquinate dehydratase; shikimate dehydrogenase^#^	(M)	S	[87]	P
At1g08510	C4	acyl-acyl carrier protein thioesterase^#^	(M)	[21]	–	T
At4g23100	C	gamma-glutamylcysteine synthetase^#^	(M)	[88]	[89]	[90]
At5g24400	C	6-phosphogluconolactonase	(M)	[91]	–	[91]
At5g52920	C4	similar to pyruvate kinase isozyme G^#^	(M)	[92]	–	[92]
At2g19450	C4	diacylglycerol O-acyltransferase / acyl CoA:diacylglycerol acyltransferase^#^	(M)	[93, 94]	[93, 94]	[95]
At1g78580	C	trehalose-6-phosphate synthase 1^#^	(M)	[96]	[97]	T
At3g10690^+^	C3	DNA gyrase subunit A family protein^#^	(PGME)	[98]	–	[98]
At3g06430^+^	NC	pentatricopeptide (PPR) repeat-containing protein^#^	(PGME)	S	–	T
At3g18110	C	pentatricopeptide (PPR) repeat-containing protein^#^	(PGME)	[61]	–	T
At3g49170	C	pentatricopeptide (PPR) repeat-containing protein^#^	(PGME)	[61]	–	T
At4g20090	C	pentatricopeptide (PPR) repeat-containing protein^#^	(PGME)	[61]	–	T
At5g27270	NC	pentatricopeptide (PPR) repeat-containing protein^#^	(PGME)	S	–	T
At1g06145	UC	similar to pentatricopeptide (PPR) repeat-containing protein^#^	(PGME)	S	–	T
At1g79350	NC	DNA-binding protein	(PGME)	S	–	T
At1g70070^+^	C3	DEAD/DEAH box helicase^#^	(PGME)	[99, 100]	–	P
At1g74970	NC	Ribosomal protein S9^#^	(PGME)	S	–	[101]
At1g14610	C	valyl-tRNA synthetase^#^	(PGME)	[56]	–	[57]
At5g02250	C4	ribonuclease II family protein^#^	(PGME)	[102]	[102]	[103]
At2g28000	C3	RuBisCO subunit binding-protein alpha subunit (Cpn60a)^ #^	(PH)	[104]	[105]	P
At1g19800	C4	Permease (TGD1, trigalactosyldiacylglycerol 1)	(T)	[106]	[107]	[106]
At4g33460	NC	ABC-type transport protein^#^	(T)	S	–	P
At2g01735	C	zinc finger (C3HC4-type RING finger) family protein^#^	(U)	S	–	T
At5g22640	C	MORN (Membrane Occupation and Recognition Nexus) repeat-containing protein^#^	(U)	S	–	P
At3g07430	C	YGGT family protein^#^	(U)	S	–	P
At1g58210	C	kinase interacting family protein, similar to kinase interacting protein 1^#^	(U)	S	–	T
At4g28210	C	Unknown	(U)	S	–	T
At1g21390	NC	Unknown	(U)	S	–	T
At1g56200	C3	Unknown	(U)	[108]	–	[108]
At5g53860	C	Unknown	(U)	S	–	P
At1g49510	C2	Unknown	(U)	S	–	T
**Genes whose disruption causes embryo lethality, but its terminal phenotype unknown (1)**						
At2g03050^*^	C	similar to the mitochondrial transcription termination factor	(PGME)	[34]	–	[34]

a Genes not listed in the SeedGenes database but reported in individual literatures are indicated with an asterisk (^*^), and those that give mutants with no viable homozygotes as reported by Myouga *et al.* [42] with a plus symbol (^+^).

b ID indicates identity confidence as defined in SeedGenes database. C, confirmed by the presence of multiple alleles causing an embryo arrest or by the genetic complementation assay; C2, having multiple null-lines with insertions in different portions of exons showing different terminal phenotypes; C2’, having multiple alleles including the ones with 5’UTR insertion causing a phenotype different from those with coding region insertions; C3, having null-mutant seeds that can germinate and develop into seedlings but not beyond; C4, having null-mutant seeds that can germinate and develop into mature plants; NC, not confirmed (only a single mutant allele with sequence information is available); UC, uncertain (insertion or mutation site not within coding region or 5' UTR and either downstream of stop codon or more than 250 bp upstream of start codon. The information of identity confidence extracted from SeedGenes database has been further updated with recent reports.

c Function is assigned based on annotation in public database (GreenPhylDB http://greenphyl.cirad.fr/cgi-bin/greenphyl.cgi as indicated with a number sign #) or individual publications. Cat, functional categories: M, metabolism; PGME, plastid gene maintenance and expression; PH, protein homeostasis; PT, protein trafficking; T, transport; U, unknown.

d References are listed for EMB (embryo deficiency), F (function), and L (localization). S, embryo-defective mutants were reported only by SeedGenes database; P, subcellular localization was confirmed only by proteomic research (compliled by PPDB [40]) but not other means; T, subcellular localization was predicted by TargetP [41] but has not been confirmed by experiments.

**Table 2.** Unambiguous Gene Expression Data Available in GeneChip for Essential Plastid-Targeted Protein-Encoding Genes

Terminal phenotype	I [Preglobular]	II [Globular]	III [Transition]	IV [Cotyledon]	Unknown
Total	19	38	15	35	1
Expression analyses available	18	33	13	30	1
Expressed at all stages of embryo^a^	11	21	7	17	0
Not detected in any stage of embryo^a^	2	2	3	6	1
Not detected in preglobular stage	4	6	3	8	1
Not detected in globular stage	6	5	3	9	1
Not detected in heart stage	3	2	3	8	1
Not detected in linear cotyledon stage	4	7	3	7	1
Not detected in mature green stage	5	11	6	11	1

Different compartments of Arabidopsis seeds were collected at different developmental stages and gene expression profile of these compartments were analyzed. For the 108 embryogenesis-essential, plastidic protein-encoding genes, 95 of them have unambiguous probe sets on Arabidopsis whole genome ATH1 GeneChip.

a The five stages at which embryo samples were taken for analyses (Fig. **1**).

**Table 3.** Genes Encoding Plastid Proteins Required for Embryogenesis whose Expression Data are Unavailable or Ambiguous on the Arabidopsis whole Genome ATH1 GeneChip

**Not covered in the Chip**
At2g04842
At3g06430
At5g22800
At5g26742
**A probe is available but shared with another gene**
At2g31530
At3g55610^a^
At4g23430
At5g10330
**Two probes are available and results are not consistent**
At5g63420
**Defined as distinct genes by AGI and locus identifier**
At1g06145
At1g21390
At3g49170
At5g03800

a Expression in embryo was reported in the reference [86].

**Table 4.** Genes Encoding Plastid Proteins Required for Embryogenesis that are Not Expressed in Embryos but in Other Seed Compartments

Gene	Terminal Phenotype	Expression in non-embryo compartment(s)
At1g19800	IV	Peripheral endosperm; Chalazal seed coat; Seed coat
At2g28880	I	Micropylar endosperm; Peripheral endosperm; Chalazal endosperm; Chalazal seed coat
At2g37920	III	Micropylar endosperm; Peripheral endosperm; Chalazal endosperm
At3g20440	III	Peripheral endosperm
At4g30580	IV	Peripheral endosperm
At4g33460	IV	Peripheral endosperm; Seed coat

Expression data for individual seed compartments is available at http://seedgenenetwork.net.
